# Infarct tissue characterization in implantable cardioverter-defibrillator recipients for primary versus secondary prevention following myocardial infarction: a study with contrast-enhancement cardiovascular magnetic resonance imaging

**DOI:** 10.1007/s10554-012-0077-6

**Published:** 2012-06-09

**Authors:** Marlon A. G. M. Olimulder, Karin Kraaier, Michel A. Galjee, Marcoen F. Scholten, Jan van Es, Lodewijk J. Wagenaar, Job van der Palen, Clemens von Birgelen

**Affiliations:** 1 Department of Cardiology, Thoraxcentrum Twente, MST, Haaksbergerstraat 55, 7513 ER Enschede, The Netherlands; 2 Department of Epidemiology, University of Twente, Enschede, The Netherlands; 3 Department of Research Methodology, Measurement and Data Analysis, University of Twente, Enschede, The Netherlands; 4 MIRA—Institute for Biomedical Technology and Technical Medicine, University of Twente, Enschede, The Netherlands

**Keywords:** Infarct tissue characteristics, Implantable cardioverter-defibrillator, Myocardial infarction, Cardiovascular magnetic resonance imaging

## Abstract

Knowledge about potential differences in infarct tissue characteristics between patients with prior life-threatening ventricular arrhythmia versus patients receiving prophylactic implantable cardioverter-defibrillator (ICD) might help to improve the current risk stratification in myocardial infarction (MI) patients who are considered for ICD implantation. In a consecutive series of (ICD) recipients for primary and secondary prevention following MI, we used contrast-enhanced (CE) cardiovascular magnetic resonance (CMR) imaging to evaluate differences in infarct tissue characteristics. Cine-CMR measurements included left ventricular end-diastolic and end-systolic volumes (EDV, ESV), left ventricular ejection fraction (LVEF), wall motion score index (WMSI), and mass. CE-CMR images were analyzed for core, peri, and total infarct size, infarct localization (according to coronary artery territory), and transmural extent. In this study, 95 ICD recipients were included. In the primary prevention group (n = 66), LVEF was lower (23 ± 9 % vs. 31 ± 14 %; *P* < 0.01), ESV and WMSI were higher (223 ± 75 ml vs. 184 ± 97 ml, *P* = 0.04, and 1.89 ± 0.52 vs. 1.47 ± 0.68; *P* < 0.01), and anterior infarct localization was more frequent (*P* = 0.02) than in the secondary prevention group (n = 29). There were no differences in infarct tissue characteristics between patients treated for primary versus secondary prevention (*P* > 0.6 for all). During 21 ± 9 months of follow-up, 3 (5 %) patients in the primary prevention group and 9 (31 %) in the secondary prevention group experienced appropriate ICD therapy for treatment of ventricular arrhythmia (*P* < 0.01). There was no difference in infarct tissue characteristics between recipients of ICD for primary versus secondary prevention, while the secondary prevention group showed a higher frequency of applied ICD therapy for ventricular arrhythmia.

## Introduction

Ventricular arrhythmia (VA) is a major cause of sudden cardiac death (SCD) in patients with prior myocardial infarction (MI) [1]. Several randomized trials have shown a beneficial effect of implantable cardioverter-defibrillator (ICD) therapy among MI patients with prior life-threatening VA (secondary prevention) [2–4]. In the setting of prophylactic ICD therapy (primary prevention), left ventricular ejection fraction (LVEF) below 35 % is considered an indication for ICD implantation. However, <25 % of these ICD recipients actually experience a life-threatening VA requiring shock therapy during median follow-up of 45.5 months [5]. Current guidelines consider a low LVEF post-MI as the most important criterion to determine a patient’s eligibility. Therefore, these guidelines appear to be suboptimal [1]. Better risk stratification is warranted to reduce the number of unnecessary device implantations, especially in the setting of primary prevention [3, 5–7].

Cardiovascular magnetic resonance (CMR) imaging in combination with the contrast-enhancement (CE) technique allows the accurate assessment of LV geometry and function as well as tissue characteristics. This permits accurate assessment of size, heterogeneity, and transmurality of the myocardial scar [8, 9]. Infarct tissue characteristics (e.g. localization, heterogeneity) [10–13] are considered potential predictors of life-threatening VAs, and could play a role in risk stratification before ICD implantation [8, 9, 14].

Previous studies demonstrated a higher occurrence of VA (and thus a higher incidence of appropriate ICD therapy) in ICD recipients for secondary prevention compared to patients who received an ICD in the setting of primary prevention [2, 15–18]. Insight into potential differences in infarct tissue characteristics between ICD recipients for primary versus secondary prevention may potentially help to improve the current practice of risk stratification in MI patients considered for ICD implantation, specifically in the primary prevention group.

Therefore, in a consecutive series of ICD recipients for primary and secondary prevention following MI, we used CE-CMR to evaluate potential differences in infarct tissue characteristics.

## Methods

### Patient population

The study was conducted at Medisch Spectrum Twente, Enschede, the Netherlands. A consecutive series of patients with prior MI, who received an ICD for primary or secondary prevention following current guidelines of the Dutch (NVVC) and European society of Cardiology (ESC) in which the LVEF was determined based on echocardiographic findings, was assessed. The referring physicians had no access to the CMR report before defining therapeutic management. Prior to ICD implantation, these patients were referred for CMR to assess left ventricular (LV) dimension and function, and after intravenous injection of gadolinium, characterization of the infarcted tissue. According to current guidelines, the patients who received ICD treatment for primary prevention had an indication based on a LVEF ≤ 35 % (majority of patients) or the presence of spontaneous ventricular tachycardia, even with a somewhat more preserved LV function. Patients were only included in the study if the MI occurred at least 1 month prior to CMR (according to the definition of a healed MI [19] and a positive infarct pattern on CE imaging was found. The study was approved by the local ethics committee and informed consent was obtained.

As the secondary prevention group (dissimilar to the primary prevention group) was not selected based on a particularly low ejection fraction, the mean LVEF of this group may be expected to be higher. To correct for this potential confounder, ICD recipients from both groups with a LVEF ≤ 35 % were separately compared. A comparable subgroup analysis in ICD recipients with a LVEF > 35 % was not performed as the limited number of patients did not permit a meaningful analysis.

### CMR data acquisition

CMR examination was performed on a 1.5-T whole body scanner (Achieva scan, Philips Medical System, Best, the Netherlands) using commercially available software. For signal-reception a five-element cardiac synergy coil was used. Electrocardiogram triggering was performed with a vector-electrocardiogram set-up. Subjects were examined in the supine position. Cine (morphologic) images in the cardiac short-axis, four-chamber, three-chamber, two-chamber long-axis, and LV outflow tract views were acquired by using fast field echo cine images. (Slice thickness 8.0 mm, repetition time 3.4 ms; echo time 1.7 ms; flip angle 60°; matrix 256 × 256).

Myocardial scar was assessed on CE multislice short- axis, long-axis and four-chamber views, obtained 10 min after intravenous bolus injection of 0.2 mmol gadolinium/kg body weight (Shering AG, Berlin, Germany). A three-dimensional Turbo Field Echo-inversion recovery T1-weighted sequence was used with the following parameters: repetition time 4.0 ms; echo time 1.3 ms; flip angle 15°; inversion time individually optimized to null myocardial signal (usually between 180 and 250 ms); matrix 157; and slice thickness 10 mm.

### CMR data analysis and definitions

CMR data were analyzed on a workstation using dedicated software (Philips MR workspace, Release 2.5.3.0; the Netherlands). Analysis was performed by reviewers blinded to clinical information.

#### LV geometry and function

Left ventricular end-diastolic and end-systolic volumes (EDV and ESV; ml), left ventricular ejection fraction (LVEF; %), and end-diastolic wall mass (EDWM; g) were calculated from contiguous short-axis loops by segmentation of endocardial and epicardial borders on each frame. Body surface area adjusted EDV (EDVi), ESV (ESVi) and EDWM (EDWMi) were also calculated.

The left ventricular wall regions were further divided into 17 segments according to a standardized myocardial segmentation model [20]. Normal wall motion was assigned a score 0, hypokinesia 1, severe hypokinesia 2, akinesia 3, and dyskinesia 4. The wall motion score index (WMSI) was calculated by dividing the sum of scores in each segment by the total number of observed segments.

#### Infarct tissue characteristics

**Fig. 1:**
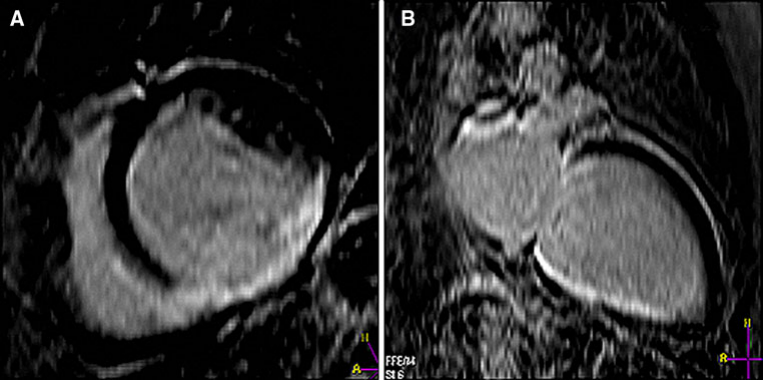
CE-CMR of a secondary prevention patient with a previous MI. **a** Short-axis view showing contrast-enhancement inferoseptal, inferior and inferolateral. **b** Long-axis view showing contrast-enhancement inferior

Infarcted myocardium was defined as the zone of hyperenhancement on the CE images, in contrast with the dark-gray signal of normal myocardium (Fig. 1). Infarct size was quantified by a semi-automatic thresholding technique with the full width at half maximum approach as previously validated [21]. After outlining the myocardial segment containing the region with high signal intensity, the maximum signal intensity region was determined. Scar was divided into an infarct core zone and a heterogeneous zone (i.e. peri-infarct zone). Infarct core was then defined as myocardium with a signal intensity ≥50 % of the maximal signal intensity. The heterogeneous zone was defined as myocardium with a signal intensity between ≥35 and <50 % of maximal signal intensity. Total scar was defined as the sum of infarct core plus heterogenous zone.

Scar tissue characteristics were further quantified according to location by use of a 17 segmental model [20]. Each segment was scored as follows: a scar score of 0 was defined normal, 1 as 1–25 % scar, 2 as 26–50 % scar, 3 as 51–75 % scar, and 4 as 76–100 % scar of the segmental area.

The *transmural extent* of myocardial scar was defined as the number of segments with a scar score 3 or 4 [22]. In addition, a segmental *regional scar score* was calculated in order to relate scar size to the territories of the three major coronary arteries as previously described in detail [20].

### Follow up and definition of events

Follow-up was performed by our outpatient clinic, including registration of the occurrence of events and survival status. Regular device interrogation was scheduled every 3–6 months. In case of any experienced event, an additional device interrogation was performed. Device therapy was classified as appropriate or inappropriate. Appropriate ICD therapy was defined as anti-tachycardia pacing and/or appropriate shock in response to ventricular tachycardia or ventricular fibrillation. For the purpose of this study, only appropriate device therapies were considered as arrhythmic events. Mortality was reported and causes of death were scored as follows: (1) myocardial infarction, (2) heart failure, (3) cerebrovascular accident, (4) carcinoma, or (5) other causes of death.

A major cardiovascular event (MACE) was defined as appropriate ICD therapy and/or death.

### Statistical analysis

Continuous variables had a normal distribution and were expressed as mean ± SD. Categorical data were expressed as frequencies and percentages. To compare the primary and secondary prevention groups, Student’s *t* test and Mann–Whitney *U* test were used to compare continuous variables, and Chi-square test and Fisher exact test were used to compare categorical variables. A survival analysis was performed to investigate if the association between infarct tissue characteristics with MACE is different among groups (ICD for primary preventions vs. ICD for secondary prevention). A *P* value < 0.05 was considered statistically significant.

## Results

### Study patients

In this study, 95 patients (64 ± 10 years old; 79 men) with a median of 141 (1–434) months after MI were examined. A total of 66 patients received an ICD for primary prevention and 29 patients for secondary prevention. Indication for secondary prevention by ICD implantation was (1) SCD in 14 patients (48 %) or (2) VT episodes in 15 patients (52 %). Time between events and ICD implantation was on average 2 weeks (median). (25th percentile 1 week, 50th percentile 2 weeks, 75th percentile weeks). In general, ICD implantation was performed shortly after CE-CMR assessment; the average (median) within 1 week, the 75th percentile within 1 week and the 90th percentile within 5.4 weeks. Demographics and baseline characteristics did not differ between groups except for diuretic usage (80 % vs. 45 %; *P* < 0.01). Patient demographics are presented in Table 1, which also shows a subgroup of patients with LVEF ≤ 35 %.

**Table 1:** Patients characteristics

	All patients			Patients with LVEF ≤ 35 %		
	Primary prevention (n = 66)	Secondary prevention (n = 29)	*P*	Primary prevention (n = 60)	Secondary prevention (n = 20)	*P*
Male sex, n	56 (85)	23 (79)	0.56	52 (87)	16 (80)	0.48
Age, years	65 ± 9	61 ± 11	0.09	66 ± 8	62 ± 9	0.08
Hypertension	24 (36)	11 (38)	1.00	22 (37)	8 (40)	1.00
Diabetes	18 (27)	5 (17)	0.43	17 (28)	3 (15)	0.25
Medication						
B-blocker	59 (89)	23 (79)	0.21	53 (88)	16 (80)	0.45
Ace inhibitor	50 (76)	20 (69)	0.62	43 (72)	16 (80)	0.57
Diuretic	53 (80)	13 (45)	<0.01	48 (80)	12 (60)	0.13
Statin	58 (88)	28 (97)	0.27	54 (90)	20 (100)	0.33
Infarct						
Single	56 (84)	24 (83)	0.87	51 (85)	17 (85)	0.67
Multiple	5 (8)	2 (7)		5 (8)	2 (10)	
Unknown	5 (8)	3 (10)		4 (7)	1 (5)	
Infarct localization			0.08			0.15
Anterior	33 (50)	10 (35)		28 (47)	6 (30)	
Nonanterior	18 (27)	14 (48)		17 (28)	9 (45)	
Both	15 (23)	5 (17)		15 (25)	5 (25)	
Infarct age, months	133 (1–434)	166 (1–426)	0.24	155 (1–434)	209 (13–426)	0.07

Continuous data are expressed as mean ± SD or median with range; and categorical data as frequencies and percentage

### CMR results

#### LV geometry and function

In the primary prevention group, LVEF (23 ± 9 % vs. 31 ± 14 %; *P* < 0.01) was significantly lower while ESVi (113 ± 39 ml vs. 91 ± 49 ml; *P* *=* 0.03) and WMSI (1.89 ± 0.52 vs. 1.47 ± 0.68; *P* < 0.01) were significantly higher than in the secondary prevention group (Table 2).

**Table 2:** CMR characteristics in all patients and in the subgroup of patients with LVEF ≤ 35 %

	All patients			Patients with LVEF ≤ 35		
	Primary prevention (n = 66)	Secondary prevention (n = 29)	*P*	Primary prevention (n = 60)	Secondary prevention (n = 20)	*P*
LV geometry and function						
EDV	284 ± 72	259 ± 91	0.16	293 ± 68	288 ± 95	0.81
EDVi	144 ± 38	129 ± 45	0.09	150 ± 34	144 ± 45	0.53
ESV	222 ± 75	184 ± 97	0.04	231 ± 69	225 ± 63	0.54
ESVi	113 ± 39	91 ± 49	0.03	119 ± 35	111 ± 47	0.43
EDWM	145 ± 37	143 ± 34	0.79	148 ± 37	141 ± 32	0.46
EDWMi	37 ± 10	36 ± 9	0.66	75 ± 18	73 ± 17	0.63
LVEF	23 ± 9	31 ± 14	<0.01	22 ± 7	23 ± 7	0.31
WMSI	1.89 ± 0.52	1.47 ± 0.68	<0.01	1.96 ± 0.48	1.76 ± 0.47	0.11
Infarct characteristics						
Infarct size-core%	12 ± 7	11 ± 9	0.62	12 ± 7	13 ± 9	0.58
Infarct size-peri%	10 ± 4	10 ± 5	0.70	10 ± 4	11 ± 5	0.53
Infarct size-total%	24 ± 10	21 ± 12	0.62	23 ± 10	24 ± 13	0.52
Infarct localization						
LAD score	1.55 ± 0.81	1.08 ± 0.84	0.02	1.54 ± 0.82	1.25 ± 0.86	0.23
RCA score	1.30 ± 0.86	1.53 ± 0.97	0.28	1.30 ± 0.85	1.62 ± 1.01	0.19
RCX score	1.10 ± 0.89	1.07 ± 0.76	0.92	1.07 ± 0.85	1.14 ± 0.86	0.80
Transmural extent	3.19 ± 2.41	2.97 ± 2.76	0.70	3.11 ± 2.46	3.55 ± 2.91	0.52

Data are presented as mean ± SD or median with range. Categorical data are presented as frequencies and percentages

*EDV* end diastolic volume, *ESV* end systolic volume, *EDWM* end diastolic wall mass, *LVEF* left ventricular ejection fraction, *WMSI* wall motion score index, *LAD* left anterior descending, *RCA* right coronary artery, *LCX* left circumflex

#### Infarct characteristics

**Fig. 2:**
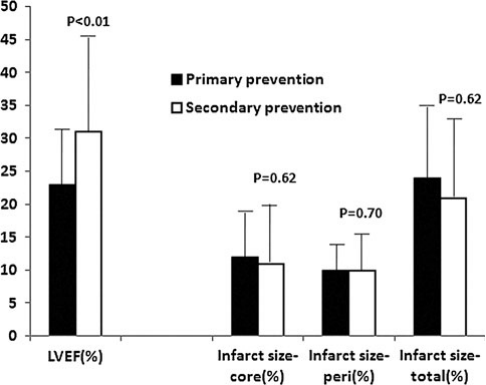
LVEF and infarct tissue characteristics (mean ± SD) in primary and secondary prevention patients. While in primary versus secondary prevention patients LVEF differed significantly, there were no significant differences in infarct tissue characteristics

There were no significant differences between size of the infarct core (12 ± 7 % vs. 11 ± 9 %; *P* = 0.62), size of the peri-infarct (10 ± 4 % vs. 10 ± 5 %; *P* = 0.70), total infarct size (24 ± 10 % vs. 21 ± 12 %; *P* = 0.62) and transmural extent (3.19 ± 2.41 vs. 2.97 ± 2.76; *P* = 0.70) of the infarct (Fig. 2). According to the regional scar score, left anterior descending scar score (1.55 ± 0.81 vs. 1.08 ± 0.84; *P* = 0.02) was significantly higher in the primary prevention group (Table 2).

### CMR results in subgroup of patients with LVEF ≤ 35 %

In order to correct for the difference in LVEF between the primary and secondary prevention group (Table 2), patients with an LVEF ≤ 35 % were included in a subanalysis. There was no difference in demographics and baseline characteristics of the remaining 60 and 20 patients, respectively. According to LV geometry and function as well as CE-CMR assessed infarct tissue characterization, no significant differences were observed between primary and secondary prevention patients with an LVEF ≤ 35 % (Table 2).

### CMR results according to infarct localization

As infarct localization differed between the primary and secondary prevention group (Tables 1 and 2), patients were stratified according to infarct localization. Between the 33 and 10 patients with anterior wall MI, respectively, there was no significant difference in LV dimensions, LV function, or infarct tissue characteristics (Table 3). Among patients with non-anterior infarct localization, the primary prevention group (n = 18) showed a lower LVEF (22 ± 9 % vs. 31 ± 14 %; *P* = 0.04) and a higher WMSI (2.10 ± 0.52 vs. 1.47 ± 0.50; *P* < 0.01) than the secondary prevention group (n = 14); but there was no significant difference in infarct tissue characteristics (Table 3).

**Table 3:** CMR characteristics in all patients stratified according to infarct localization

	Anterior infarction			Nonanterior infarction		
	Primary prevention (n = 33)	Secondary prevention (n = 10)	*P*	Primary prevention (n = 18)	Secondary prevention (n = 14)	*P*
LV geometry and function						
EDV	282 ± 79	275 ± 106	0.83	286 ± 67	250 ± 90	0.20
EDVi	144 ± 40	135 ± 50	0.56	149 ± 35	126 ± 48	0.17
ESV	218 ± 81	192 ± 119	0.43	225 ± 75	180 ± 99	0.15
ESVi	111 ± 41	95 ± 58	0.31	117 ± 40	90 ± 53	0.15
EDWM	137 ± 42	140 ± 34	0.83	158 ± 29	145 ± 32	0.24
EDWMi	71 ± 21	69 ± 15	0.81	82 ± 14	75 ± 19	0.26
LVEF	25 ± 9	32 ± 17	0.22	22 ± 9	31 ± 14	0.04
WMSI	1.73 ± 0.52	1.51 ± 0.89	0.32	2.04 ± 0.56	1.36 ± 0.62	<0.01
Infarct characteristics						
Infarct size-core%	12 ± 6	11 ± 9	0.47	11 ± 6	10 ± 7	0.62
Infarct size-peri%	11 ± 4	11 ± 6	0.77	9 ± 3	9 ± 5	0.80
Infarct size-total%	23 ± 9	22 ± 14	0.71	21 ± 9	19 ± 11	0.65
Transmural extent	3.29 ± 2.22	2.80 ± 2.90	0.58	3.60 ± 0.47	2.79 ± 2.69	0.40

See legend of Table 2 for details

### Follow-up

During 21 ± 9 months of follow-up, 4 patients in the primary prevention group and 4 patients in the secondary prevention group died, respectively. All 8 patients died on heart failure. The frequency of appropriate ICD therapy differed significantly between the primary and secondary prevention group (3/66 (5 %) vs. 9/29 (31 %); *P* < 0.01). In the primary prevention group, only appropriate shock therapy (2 on VF, 1 on VT) was delivered, while in the secondary prevention group both appropriate shock therapy (n = 3; all on VT) and antitachycardia pacing (n = 6) were delivered. All but 2 patients with appropriate ICD therapy (both secondary prevention) had a ≤35 %.

Patients with appropriate ICD therapy did not differ from patients without event in peri-infarct size (8.83 ± 3.20 % vs. 10.51 ± 4.39 %; *P* = 0.20), but core infarct size was smaller in patients with events (8.00 ± 4.93 % vs. 12.58 ± 7.37 %; *P* = 0.040).

The association between infarct tissue characteristics with MACE did not significantly differ among groups (ICD for primary prevention vs. ICD for secondary prevention) (*P* = 0.25–0.91).

## Discussion

The implantation of ICD in MI patients provides protection from SCD following VA. When current guidelines are followed, <1 out of 4 primary prevention ICD recipients experiences actual life-threatening VA requiring shock therapy during a follow-up period of almost 4 years [5]. This shows that there may be some room for improvement in the selection of ICD candidates in the setting of primary prevention.

The infarct core and heterogeneous zone, as well as presence of transmural infarction may serve as an anatomic pathway for reentry, and consequently, the occurrence of VA [10–13, 23, 24]. In this respect, it has recently been demonstrated that a larger size of infarct heterogeneity is related to increased ventricular irritability by programmed electrical stimulation as well as spontaneous VA [8, 9].

While there was a difference in frequency of applied ICD therapy between primary and secondary prevention patients in our study, there was no difference in the size of the infarct tissue characteristics between these two subpopulations of patients. These findings may question the importance of the size of infarct tissue characteristics as a predictor of life-threatening VA [25]. However, size of infarct tissue characteristics is not really all that matters, as it has been demonstrated that a substantial portion of tachycardia originates from reentry occurring in a very small circuit extending just over a few millimeters [26]. Other factors than anatomic substrate may interfere with the risk of VA in the setting of MI; an example may be genetic factors. In this respect, recently, a genome-wide association study identified in patients with a first MI a gene locus prone for ventricular fibrillation [27].

In the primary prevention group, we found a substantially larger amount of MI tissue in the anterior wall of the LV during CMR assessment. Several clinical studies observed that patients with anterior MI usually have a worse LVEF [28]. The larger amount of anterior MI in the primary prevention group may thus actually be expected, as LVEF below 35 % is used as a major risk stratifier for primary prevention with ICD, according to current guidelines. In addition, in our primary prevention group (*P* < 0.01) there was a higher use of diuretics for symptomatic treatment of heart failure. On the other hand, secondary prevention patients—patients who already had a life-threatening VA in the past—showed more appropriate ICD therapies during follow-up, as may be expected based on the difference in indication. Thus, there must be other factors than the studied CMR characteristics involved to make the myocardium prone to the development of life-threatening VA.

With current clinically applied CE-CMR technology, spatial resolution imposes constraints on what type of tissue is concealed within the peri-infarct zone, characterized by intermediate signal intensities [29]. High-resolution CE-CMR imaging with 1,000-fold higher resolution than clinical scans may bear the potential to obtain further insights in an experimental setting [30]. There is a lack of well-defined gold standard formula for the assessment of infarcted myocardium. Partial volume effects and blurred images by cardiac motion during image acquisition may lead to a relative increase of signal intensity in pixels of the border zone of infarcted compared to remote myocardium, which may lead to an overestimation of the total scar score. Initial visual assessment, manual tracing of endo and epicardial contours, visual identification of the region of interest with maximum signal intensity, and visual check for erroneous inclusion of other regions with high signal intensity (e.g. in/folding or motion artefacts, fat, or pericardial effusion) [31] require experience and involve a certain degree of interpretation. The subjectivity involved can only be minimized by an optimized training of experienced analysts. Finally, signal intensity analysis with current CE-CMR techniques do not incorporate areas of microvascular obstruction, which are hypo-enhanced in CE-CMR [31], which may lead to underestimation of infarct size. As only one patient of the present patient population showed microvascular obstruction, the microvascular incorporated to obstruction area was not the infarct core.

While CMR is the gold standard for the assessment of LV function, myocardial viability, extent and transmurality of scar, our findings suggest that infarct tissue analysis with the CE-CMR technique that is currently applied in clinical practice does not appear to have the potential to improve the current practice of risk stratification in MI patients considered for ICD implantation.

## Limitations

Our study comprises a limited number of patients; nevertheless, this represents a consecutive series of patients examined with CE-CMR for that indication. While the secondary outcome of defibrillator shocks was prospectively collected and analyzed, the primary comparison of CE-CMR image characteristics was based on a cross-sectional approach. In the light of the duration of clinical follow-up of 21 ± 9 months, event rates in subgroups should be interpreted carefully. In addition, primary aim of the present study was the assessment of potential differences in infarct tissue characteristics between ICD recipients for primary versus secondary prevention.

## Conclusion

There was no difference in infarct tissue characteristics between recipients of ICD for primary versus secondary prevention, while the secondary prevention group showed a higher frequency of applied ICD therapy for ventricular arrhythmia.

## Abbreviations

VA: Ventricular arrhythmia
SCD: Sudden cardiac death
MI: Myocardial infarction
ICD: Implantable cardioverter-defibrillator
LVEF: Left ventricular ejection fraction
CMR: Cardiovascular magnetic resonance
CE: Contrast enhancement
EDV: End-diastolic volume
ESV: End-systolic volume
EDWM: End-diastolic wall mass
WMSI: Wall motion score index
EDWT: End-diastolic wall thickness
MACE: Major cardiovascular event
